# Parameter Identification of Multispan Rigid Frames Using a Stiffness Separation Method

**DOI:** 10.3390/s24061884

**Published:** 2024-03-15

**Authors:** Feng Xiao, Yu Yan, Xiangwei Meng, Yuxue Mao, Gang S. Chen

**Affiliations:** 1 Department of Civil Engineering, Nanjing University of Science and Technology, Nanjing 210094, China; yy915160352@njust.edu.cn (Y.Y.); mxw@njust.edu.cn (X.M.); maoyuxue@njust.edu.cn (Y.M.); 2 College of Engineering and Computer Sciences, Marshall University, Huntington, WV 25755, USA; chenga@marshall.edu

**Keywords:** parameter identification, multispan rigid frame, stiffness separation method, static response, joint and member damage

## Abstract

Identifying the parameters of multispan rigid frames is challenging because of their complex structures and large computational workloads. This paper presents a stiffness separation method for the static response parameter identification of multispan rigid frames. The stiffness separation method segments the global stiffness matrix of the overall structure into the stiffness matrices of its substructures, which are to be computed, thereby reducing the computational workload and improving the efficiency of parameter identification. Loads can be applied individually to each separate substructure, thereby guaranteeing obvious local static responses. The veracity and efficacy of the proposed methodology are substantiated by applying it to three- and eight-span continuous rigid frame structures. The findings indicate that the proposed approach significantly enhances the efficiency of parameter identification for multispan rigid frames.

## 1. Introduction

Frame structures, particularly those related to civil engineering, are widely used in various structural configurations such as buildings and bridges [1]. The area of continuous rigid-frame bridges has been investigated [2,3,4,5,6,7]. Yoshikawa et al. [5] investigated the seismic design and construction techniques of the Benten Viaduct, which is a continuous rigid-frame bridge featuring 19 spans. Zhou et al. [6] proposed a unified calculation model for the longitudinal fundamental frequency of continuous rigid-frame bridges and validated its applicability by performing experiments on a continuous rigid-frame bridge in Shaanxi Province. To examine the characteristics of additional forces on structures, Liu et al. [7] numerically investigated the continuously welded rails of a rigid-frame bridge of the Fuzhou–Xiamen High-Speed Railway. Among the different types of steel-frame structures, single-story industrial steel building structures are the most ubiquitous [1,8,9,10]. Scozzese et al. [9] investigated seismic nonstructural damage and proposed a method that enabled the assessment of the severity and scope of nonstructural damage in single-story industrial steel structures. Şakar et al. [1] used the finite element method and various research methods to analyze the responses of multispan frames subjected to periodic loading. However, these frames are susceptible to damage due to aging, changes in load characteristics, changes in environmental influences, and unforeseen catastrophic events such as floods and earthquakes [11,12]. Unanticipated structural failures can result in catastrophic consequences, e.g., a loss of life, economic adversity, and societal repercussions. Thus, the detection of structural damage must be prioritized, particularly during its early stages, to prevent abrupt failures and improve the safety and reliability of structures [13,14].

The structural health monitoring of these structures can be achieved through parameter identification, which is a mathematical approach that uses the errors between estimated and experimental values. Parameter identification attempts to correlate the changes in the test data with the changes in structural elemental properties. Additionally, it aims to establish a correlation between the variations in the test data and the changes in the elemental properties of a structure [13,15]. Changes in certain parameters, such as the cross-sectional area, moment of inertia, elastic modulus, and stiffness, occur because of structural damage, and thus consequently affect the static and dynamic properties of the structure, including its displacement, strain, mode shape, and natural frequency.

Static and dynamic parameter identification methods are two distinct approaches used in the field [16,17,18,19,20,21]. Some researchers have made remarkable progress in the field of structural damage identification using static responses. Sanayei et al. [22,23] focused on structural parameter identification and damage assessment based on static responses, developed formulas to estimate the structural parameters from static strain, and conducted a nondestructive inspection of structures. Xiao et al. [24] employed static responses to identify the damage in truss structures. Zhu et al. [25] proposed a method for detecting structural damage using the influence line of a sensor and an empirical Bayesian threshold estimator. This approach utilizes a quasi-static displacement influence line to obtain displacement readings and deduce the load effects on a bridge. The effectiveness of this method was demonstrated through numerical simulations and field tests conducted on bridges. Augusto et al. [26] proposed a novel parameter identification method and algorithm that utilizes structural optimization concepts to accurately identify the stiffness in linear elastic models of civil structures. Numerical examples involving a 10-bar truss structure and a two-bay, two-story moment frame have demonstrated the effectiveness of the algorithm in correctly identifying stiffness parameters. Kourehli et al. [27] proposed a novel approach for detecting and estimating structural damage using the incomplete static response data of a damaged structure and applying the least-squares support vector machine method. The approach was applied to structures including a plane-rigid bridge, a four-span continuous beam, and a four-story plane frame with multiple damages. Parameter identification and assessment methods based on static responses primarily compare structural displacement, rotation angle, and strain under static loads for damage detection. Static test data are relatively unaffected by environmental factors because of their loading regime, thus resulting in relatively stable test results. Vibration-based methods are typically affected by environmental factors. In contrast, static damage indicators are more sensitive to local damage [18].

For damage identification in multispan rigid frame structures, Zhang et al. [28] proposed an alternative method based on free-wave characteristics for model updating. This method was used to calibrate a finite element model of the K032 viaduct on the A11 highway in Bruges, Belgium. Considering the engineering background of the Renyihe Bridge, which is a concrete continuous rigid-frame bridge, Cheng et al. [29] introduced a practical approach that relied on updating a dynamic finite element model. Fan et al. [18] focused on damage identification in tied-arch bridge hangers. Practical solutions derived from mechanical models and the finite element verification of displacement difference influence lines were employed in their study. Deng et al. [30] developed a damage identification method that relied on the correlation between the probability distribution of quasi-static response data. By monitoring the strain and tension of long-span bridge structures, the proposed method was validated and found to exhibit accurate and robust performances in identifying the damage to bridge structures. Liu et al. [31] conducted a local reliability analysis of a large-span rigid-frame bridge based on strain monitoring using a long-term structural health monitoring (SHM) system. 

Three primary challenges are encountered when addressing large-scale structures. First, the analytical models of such structures encompass a significant number of degrees of freedom (DOFs), thus necessitating substantial storage space for the resulting mass matrix and stiffness matrix. Second, extracting the eigensolutions and sensitivity matrices from their mass and stiffness matrices requires considerable computational effort as repetitive calculations are required. Finally, the optimization process may be disrupted due to the extensive number of parameters that need to be updated in a large-scale structure [32,33,34].

To address the difficulties in analyzing large-scale structures, a stiffness separation method [24,33] is employed in this study for damage identification. This method has several advantages. First, it enables an independent or concurrent analysis of substructures. Second, by analyzing the substructures instead of the overall structure, the computational difficulty is reduced, thus resulting in fewer iterations being required to optimize the values, as well as improved computational efficiency. Additionally, loads can be applied separately to each substructure, thus guaranteeing obvious local static responses. In this technique, a large-scale structure is partitioned into smaller, manageable substructures, and each substructure is independently analyzed to obtain its specific solution. 

This paper introduces an approach to partitioning high-order global stiffness matrices into lower-order matrices for the analysis of multispan rigid frames. In this method, the substructures are separated from the whole structure using static responses. The objective function is established based on measured and analytical displacements, and then the function is optimized to identify the unknown parameters. This method enables non-destructive static parameter identification for large-scale structures. Additionally, it enhances the accuracy of parameter identification by achieving evident local static responses in the structure. Moreover, it simplifies the objective equation by reducing the number of unknown parameters to be identified and improves the efficiency of parameter identification. The effectiveness and accuracy of this method are demonstrated based on two examples of multispan continuous rigid frames.

## 2. Formulation for Parameter Identification

This section presents a parameter identification method based on static responses, where the partial physical properties of a structure are defined as unknown parameters to determine the presence of damage within the structure.

### 2.1. Modeling of Structural Frame Elements

**Figure 1:**
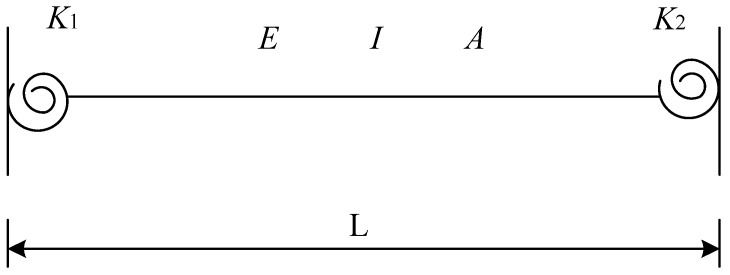
Modeling of 2D beam element with semi-rigid connections.

Rigid frames may have member and joint damage. Damage to frame elements results in reduced stiffness. Therefore, reductions in the cross-sectional area and moment of inertia of the member can be used to represent damage [22,35]. Joint damage includes beam–column joint and column base damage. To identify joint damage in rigid frame structures, the zero-length rotational spring at the end of the beam element can be used to represent the rotational stiffness of a beam-to-column connection [36,37] or a column base connection [38,39] in a frame structure. The joint fixity factor can be determined by this rotational stiffness, which ranges from 0 to 1, whereas joint damage can be represented by a reduction in the fixity factor. A two-dimensional (2D) beam element with semi-rigid connections is shown in Figure 1.

The rotational stiffness values at the different ends of the element are denoted as *K*_1_ and *K*_2_. Here, *E*, *I*, and *A* represent the modulus of elasticity, moment of inertia, and cross-sectional area, respectively, while *L* indicates the length of the member. Equation (1) defines the relationship between the ended fixity factor (*γ_j_*) and the parameters *E*, *I*, *L*, and *K*.

(1) $$\gamma_{j}=\frac{1}{1+\left(\frac{3EI/L}{K_{j}}\right)},\;j=1,\;2$$

The fixity factor value ranges from 0 to 1. *γ_j_* = 0 indicates a completely pinned connection, whereas *γ_j_* = 1 indicates a fully rigid connection. In this study, the severity of joint damage was simulated by reducing the fixity factors of the joints [40]. Equation (2) presents the 2D stiffness matrix of a semi-rigid frame member based on the Euler–Bernoulli beam theory [41].

(2) $$k^{\prime }=\left[\begin{array}{cc} \begin{array}{ccc} \frac{EA}{L} & 0 & 0\\ \; & \frac{12EI}{L^{3}}(\frac{n_{1}}{n_{7}}) & \frac{6EI}{L^{2}}(\frac{n_{2}}{n_{7}})\\ \; & \; & \frac{4EI}{L}(\frac{n_{3}}{n_{7}}) \end{array} & \begin{array}{ccc} -\frac{EA}{L} & 0 & 0\\ 0 & -\frac{12EI}{L^{3}}(\frac{n_{1}}{n_{7}}) & \frac{6EI}{L^{2}}(\frac{n_{6}}{n_{7}})\\ 0 & -\frac{6EI}{L^{2}}(\frac{n_{2}}{n_{7}}) & \frac{2EI}{L}(\frac{n_{5}}{n_{7}}) \end{array}\\ \begin{array}{ccc} \; & \; & \;\\ \; & \; & \;\\ \; & Sym & \; \end{array} & \begin{array}{ccc} \frac{EA}{L} & 0 & 0\\ \; & \frac{12EI}{L^{3}}(\frac{n_{1}}{n_{7}}) & -\frac{6EI}{L^{2}}(\frac{n_{6}}{n_{7}})\\ \; & \; & \frac{4EI}{L}(\frac{n_{4}}{n_{7}}) \end{array} \end{array}\right]$$

where parameter $n_{i}$ is defined using joint fixity factors *γ*_1_ and *γ*_2_ as follows:

(3) $$n_{1}=\gamma_{1}+\gamma_{2}+\gamma_{1}\gamma_{2}\;\;n_{2}={2\gamma }_{1}+\gamma_{1}\gamma_{2}\;n_{3}={3\gamma }_{1}\;\;n_{4}=3\gamma_{2}\;\;n_{5}=3\gamma_{1}\gamma_{2}\;n_{6}=2\gamma_{2}+\gamma_{1}\gamma_{2}\;\;n_{7}=4-\gamma_{1}\gamma_{2}$$

### 2.2. Objective Function

The parameter-identification algorithm defines the discrepancy between the analytical and measured displacements using an objective function. The unknown parameters can be obtained by minimizing the objective function. In this method, static responses are obtained by applying loads to the structure. The damage condition of the structural components is determined based on the optimization of the objective function. The stiffness method can be used to calculate the analytical displacement. The relationship between the structural stiffness matrix, displacement, and force can be expressed as follows:

(4) Q=KD

where ***Q*** represents the global force; ***K*** represents the global stiffness matrix of the entire structure, which can be obtained by assembling the member stiffness matrix $k^{\prime }$ using global coordinates; and ***D*** represents the global displacements. The objective function for parameter identification can be expressed as the difference between the analytical and measured displacements. The objective function can be expressed as shown in Equation (5).

(5) $$f=\sum_{i=1}^{n}{\left(D_{m}^{i}-D_{a}^{i}\right)}^{2}$$

In Equation (5), $D_{m}^{i}$ stands for the *i*th measured displacement, $D_{a}^{i}$ denotes the corresponding *i*th analytical displacement, and *n* is the total number of measured nodal displacements. The measured and analytical displacements can be determined by solving Equation (4). The objective function can be minimized to obtain the unknown parameters.

### 2.3. Result Analysis

The mean relative error (MRE) can be employed to evaluate the accuracy and precision of an estimation method [42]. It quantifies the average percentage difference between identified and actual values. The MRE is expressed as follows:

(6) $$\mathrm{MRE}=\frac{1}{N}\sum_{i=1}^{N}\left(\frac{\left|p_{i}-p_{i}^{*}\right|}{p_{i}}\right)$$

where *N* is the number of parameters for the damaged joints and members. In this study, $p_{i}$ is the *i*th actual value and $p_{i}^{*}$ is the *i*th optimal value of this parameter, obtained by optimizing the objective function. In addition, all objective functions were solved using the Nelder–Mead method in this study. The Nelder–Mead method is an efficient direct search method that optimizes the response function by comparing function values [43].

## 3. Parameter Identification of a Three-Span Single-Layer Rigid Frame

**Figure 2:**
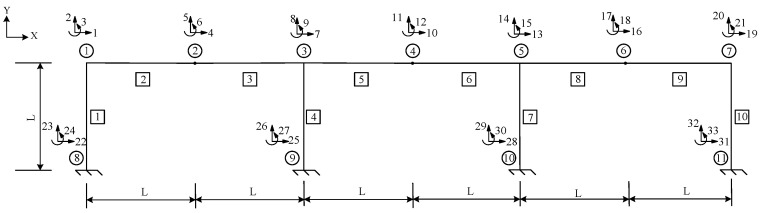
Three-span single-layer rigid frame.

**Figure 3:**
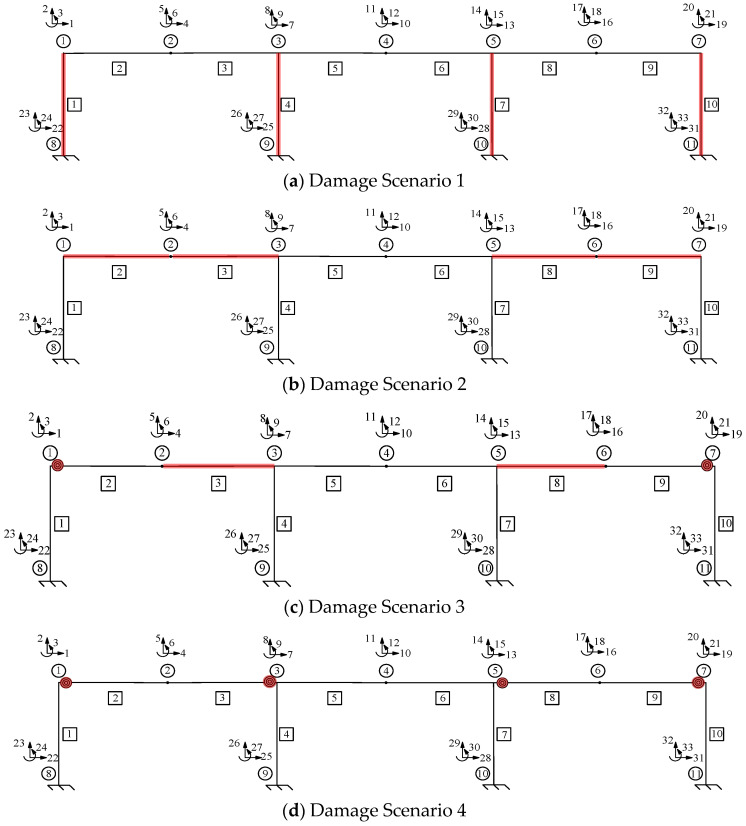
Four different damage scenarios of a three-span single-layer rigid frame.

Figure 2 presents a three-span single-layer rigid frame, which shows the number of elements within the boxes, the number of nodes within the circles, and the DOFs of each node, represented by the number next to the arrows. Assuming that the node number is *s*, the X direction, Y direction, and rotational DOFs are $\left(3\times s\right)-2$, (3×s)−1, and 3×s, respectively. The modulus of elasticity is 206 GPa. For the “as-built” conditions, all the members feature the same cross-sectional area *A* = 5.6 × 10^−3^ m^2^, *I* = 2.779 × 10^−5^ m^4^, *L* = 4 m, and a fixity factor of 1. The “as-is” conditions, which are unknown, need to be determined when the rigid frame is damaged. To prove the accuracy and feasibility of the method described above, four different damage scenarios were assumed for the structure shown in Figure 2, and a damage diagram of them is shown in Figure 3. The section shown in red indicates the damage’s location on the rigid frame. Table 1 lists the parameters that need to be identified in the four types of damage scenarios and the corresponding values of each parameter. The units for *A* and *I* are m^2^ and m^4^, respectively. However, these values are unknown during the identification process. Hence, the parameters of the rigid frame were identified based on different damage scenarios.

The four types of damage scenarios were as follows: (1) column damage, (2) beam damage, (3) beam and joint damage, and (4) joint damage. Damage Scenario 1 is used as an example herein to illustrate the identification process.

**Figure 4:**
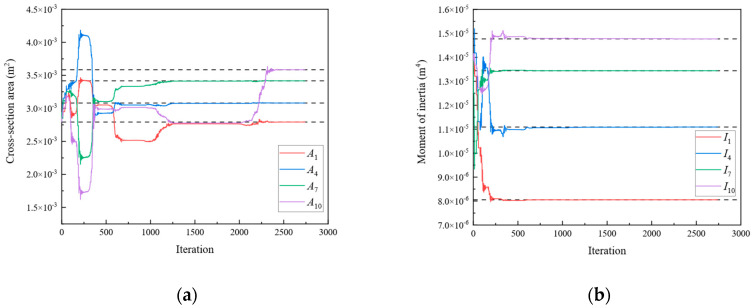
The variation of the parameters of the rigid frame with the number of iterations under Damage Scenario 1: (**a**) the cross-sectional area; (**b**) the moment of inertia.

In Scenario 1, we assumed that the locations of the damage were elements 1, 4, 7, and 10 of Figure 3a. The values of the “as-is” cross-section area and moment of inertia are shown in Table 1, which are unknown and need to be identified. To obtain the response of the rigid frame structure, forces of 50 and −50 kN were applied to 10 and 11 DOFs, respectively. For the rigid frame, $D_{m}^{i}$ was obtained through the displacement responses at 1, 2, 4, 5, 7, 8, 10, 11, 13, 14, 16, 17, 19, and 20 DOFs. In this study, the measured displacements are calculated using the direct stiffness method. The member stiffness matrix k′ was obtained using Equation (2). The formulas described in Section 2 were employed to calculate the analytical displacements $D_{a}^{i}$ for the same DOFs. Subsequently, the objective function was established, and optimization was performed. In Damage Scenario 1, eight parameters for the four damaged members need to be identified simultaneously using the established objective equation. In this study, the starting points of the cross-section area and moment of inertia variables were set at the midpoint of the “as-built” condition. Figure 4 shows the parameter identification results for the rigid frame in Damage Scenario 1. The dotted lines in the diagram represent the “as-is” values of each parameter. After 2738 iterations, the optimal values matched the “as-is” values, and the results converged. The results demonstrate that the optimized parameter values exhibit a negligible error when compared with the actual values. Similarly, Damage Scenarios 2, 3, and 4 can be identified using the proposed method. The loads applied in Damage Scenarios 2 and 4 were the same as those in Damage Scenario 1. Under Damage Scenario 3, forces of 50, −50, and −50 kN were applied under 1, 11, and 17 DOFs, respectively. Because the fixity factor ranged from 0 to 1, the starting point for the fixity factor was set to 0.5. 

**Figure 5:**
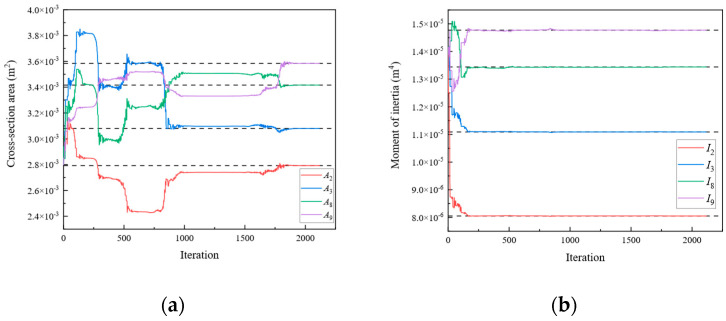
The variation of the parameters of the rigid frame with the number of iterations under Damage Scenario 2: (**a**) the cross-sectional area; (**b**) the moment of inertia.

**Figure 6:**
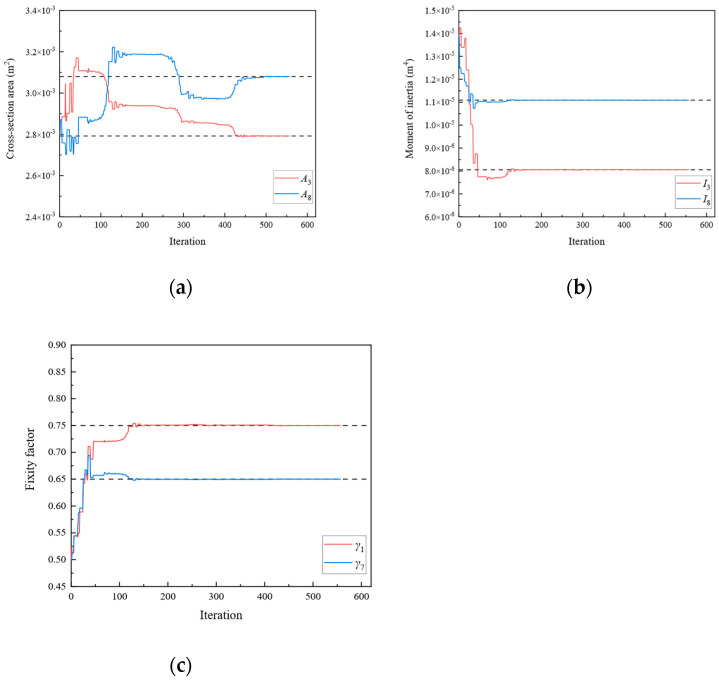
The variation of the parameters of the rigid frame with the number of iterations under Damage Scenario 3: (**a**) the cross-sectional area; (**b**) the moment of inertia; (**c**) the fixity factor.

**Figure 7:**
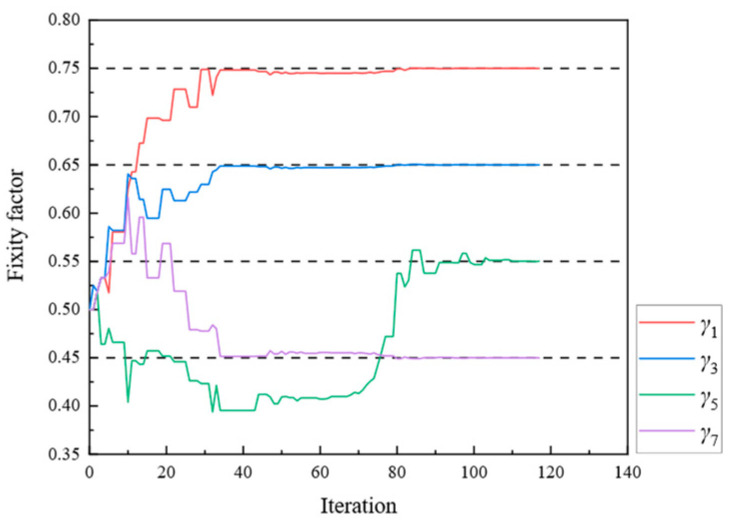
The variation of the parameters of the rigid frame with the number of iterations under Damage Scenario 4: the fixity factors.

Figure 5, Figure 6 and Figure 7 present the parameter iteration results for the rigid frame under Damage Scenarios 2–4, respectively. In Damage Scenarios 2, 3, and 4, the objective function converged after 2118, 557, and 117 iterations, respectively. Table 2 shows a comparison of the iteration steps for the different damage scenarios.

According to Table 2, the damage locations and whether the damaged members or joints affect the target equation result in different identification steps. Although Damage Scenarios 1 and 2 only involve damage to the members, their different damage locations resulted in different iteration steps. In contrast, Damage Scenarios 3 and 4 involved joint damage, and the iteration steps were reduced because one damaged member introduced two variables. However, one damaged joint only introduced one variable. Thus, the total number of variables in Damage Scenarios 3 and 4 is different from those in Damage Scenarios 1 and 2. The increased unknown parameter number resulted in a higher number of iteration steps for the objective function. However, the iterative values closely matched the actual values, with negligible errors. This indicates the effectiveness and accuracy of the parameter identification method proposed in Section 2.

## 4. Parameter Identification Using the Stiffness Separation Method

This section describes the parameter identification process for rigid frames using the stiffness separation method. 

### 4.1. Formulas of the Stiffness Separation Method

First, numbers were assigned to the joints and DOFs of the overall structure. Subsequently, the stiffness matrix of the entire structure was obtained using the method described in Section 2. Assuming that the structure has *n* degrees of freedom, the static displacement matrix is denoted as ***D***, the global stiffness matrix of the overall structure is denoted as ***K***, and the external load matrix acting on the entire structure is denoted as ***Q***.

(7) $$D={\left[D_{1},D_{2}{,\ldots ,D}_{n}\right]}^{T}\;Q={\left[Q_{1},Q_{2}{,\ldots ,Q}_{n}\right]}^{T}\;K=\left[\begin{array}{ccc} \begin{array}{cc} k_{11} & k_{12}\\ k_{21} & k_{22} \end{array} & \cdots & \begin{array}{c} k_{1n}\\ k_{2n} \end{array}\\ \vdots & \ddots & \vdots \\ \begin{array}{cc} k_{n1} & k_{n2} \end{array} & \cdots & k_{nn} \end{array}\right]$$

Similarly, the DOFs numbering of the substructure is separate from the overall structure, and the displacements at different positions are labeled according to the DOFs’ numbers. Thus, the number of unknown displacements in the separated substructure is denoted as *p* and the number of nonzero displacements in the substructure is denoted as *m*. Zero displacement can be identified based on the boundary conditions. Assume that vector ***B*** contains the DOFs corresponding to the unknown displacements in the substructure, sorted in ascending order. Vector ***U*** represents the DOFs corresponding to the nonzero displacements in the substructure, which are sorted in ascending order. The elements in ***K****_p×m_*, ***D****_m_*, and ***Q****_p_* are rearranged and composed by extracting the elements from ***K***, ***D***, and ***Q***, respectively, based on the DOF of the substructure. ***K****_p×m_* is the substiffness matrix, and the column matrix ***D****_m_* represents the nonzero displacements in the substructure, sorted in ascending order based on their corresponding DOFs. Similarly, ***Q****_p_* represents the column matrix of the external loads, where the elements in the column matrix are arranged in ascending order based on the DOFs corresponding to the unknown displacements.

(8) $$B=[b_{1},b_{2},\ldots b_{p}]\;U=[u_{1},u_{2},\ldots u_{m}]\;D_{m}={\left[D_{u_{1}},D_{u_{2}}{,\ldots ,D}_{u_{m}}\right]}^{T}\;Q_{p}={\left[Q_{b_{1}},Q_{b_{2}}{,\ldots ,Q}_{b_{p}}\right]}^{T}\;K_{p\times m}=\left[\begin{array}{ccc} \begin{array}{cc} k_{b_{1},u_{1}} & k_{b_{1},u_{2}}\\ k_{b_{2},u_{1}} & k_{b_{2},u_{2}} \end{array} & \cdots & \begin{array}{c} k_{b_{1},u_{m}}\\ k_{b_{2},u_{m}} \end{array}\\ \vdots & \ddots & \vdots \\ \begin{array}{cc} k_{b_{p},u_{1}} & k_{b_{p},u_{2}} \end{array} & \cdots & k_{b_{p},u_{m}} \end{array}\right]$$

The substiffness relationship between the forces and displacements of the substructure can be derived by extracting the elements from the global stiffness matrix as follows:

(9) $$Q_{p}=K_{p\times m}D_{m}$$

Because ***K****_p×m_* represents the substiffness matrix containing the unknown parameters to be identified, the analytical displacements with unknown parameters can be obtained by solving Equation (9). Subsequently, an objective equation is formulated to relate the analytical displacements to their corresponding measured displacements. Eventually, the values of the unknown parameters are determined via optimization and by solving the objective equation. The unknown parameters in this context refer to *A*, *I*, and *γ*.

### 4.2. Parameter Identification Example

**Figure 8:**
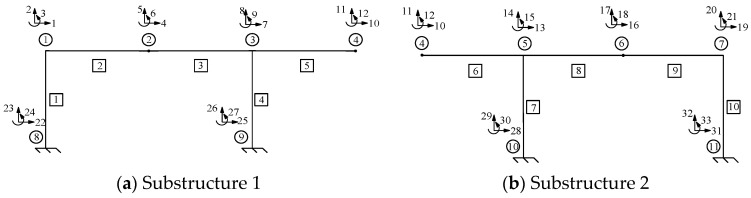
Schematic diagram of the rigid frame’s substructures.

The rigid frame structure shown in Figure 2 was analyzed. The structure was segmented into two substructures: Substructures 1 and 2. A diagram of the segmented structure is shown in Figure 8. The four different damage scenarios from Section 3 were applied, in addition to the same load conditions as those described in Section 3, to investigate the advantages of the stiffness separation method. 

In those four damage scenarios, Node 4 separates the entire rigid frame structure into two substructures, and the measured displacements are located at 10, 11, and 12 DOFs. Substructures 1 and 2 were extracted from the stiffness matrix of the overall structure. Because the displacements under 10, 11, and 12 DOFs have been measured, the analytical displacements of Substructures 1 and 2 were determined independently. The complex problem was simplified into a straightforward one by establishing boundary conditions for these substructures. Subsequently, the objective function of Substructure 1 was established based on its measured displacements under 1, 2, 4, 5, 7, and 8 DOFs, as well as their corresponding analytical displacements. Similarly, the objective function of Substructure 2 was derived based on its measured displacements under 13, 14, 16, 17, 19, and 20 DOFs, as well as their corresponding analytical displacements. Next, the objective functions were optimized to obtain the values of the unknown parameters.

**Figure 9:**
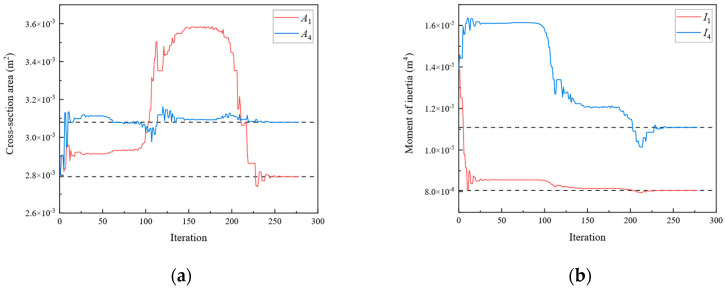
The variation of the parameters of Substructure 1 of the rigid frame with the number of iterations under Damage Scenario 1: (**a**) the cross-sectional area; (**b**) the moment of inertia.

**Figure 10:**
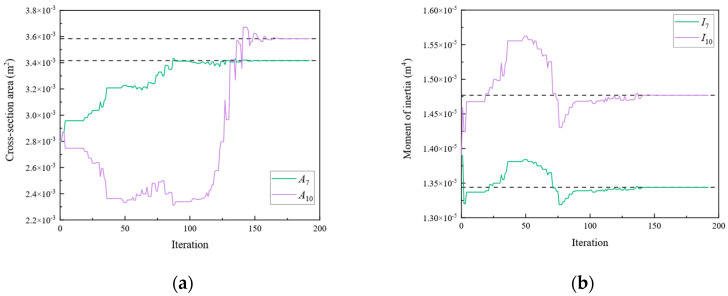
The variation of the parameters of Substructure 2 of the rigid frame with the number of iterations under Damage Scenario 1: (**a**) the cross-sectional area; (**b**) the moment of inertia.

**Figure 11:**
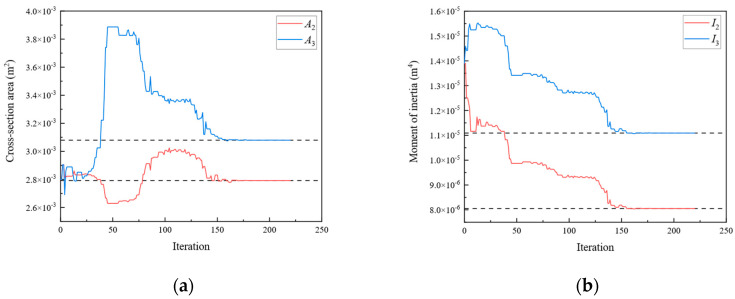
The variation of the parameters of Substructure 1 of the rigid frame with the number of iterations under Damage Scenario 2: (**a**) the cross-sectional area; (**b**) the moment of inertia.

**Figure 12:**
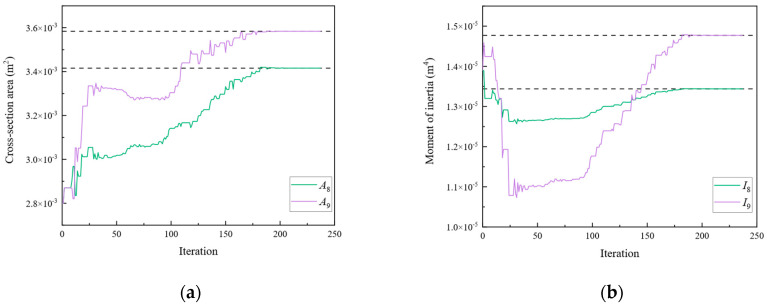
The variation of the parameters of Substructure 2 of the rigid frame with the number of iterations under Damage Scenario 2: (**a**) the cross-sectional area; (**b**) the moment of inertia.

**Figure 13:**
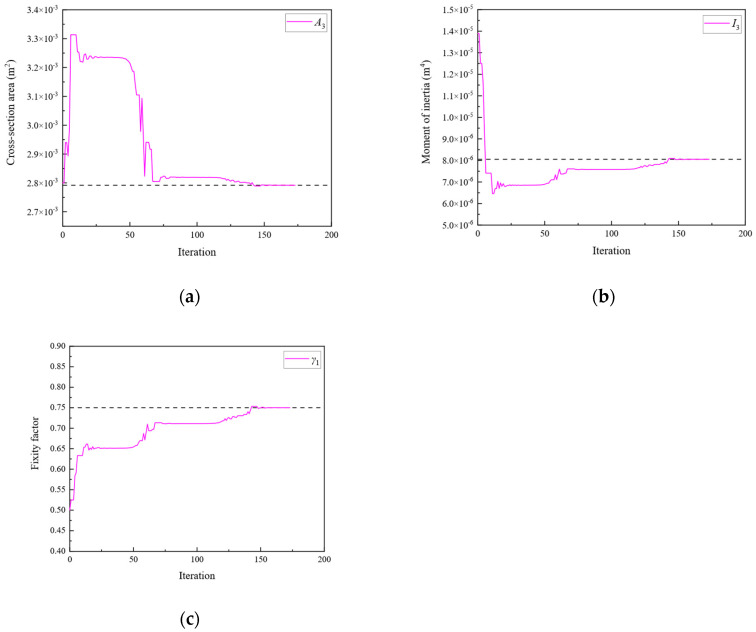
The variation of the parameters of Substructure 1 of the rigid frame with the number of iterations under Damage Scenario 3: (**a**) the cross-sectional area; (**b**) the moment of inertia; (**c**) the fixity factor.

**Figure 14:**
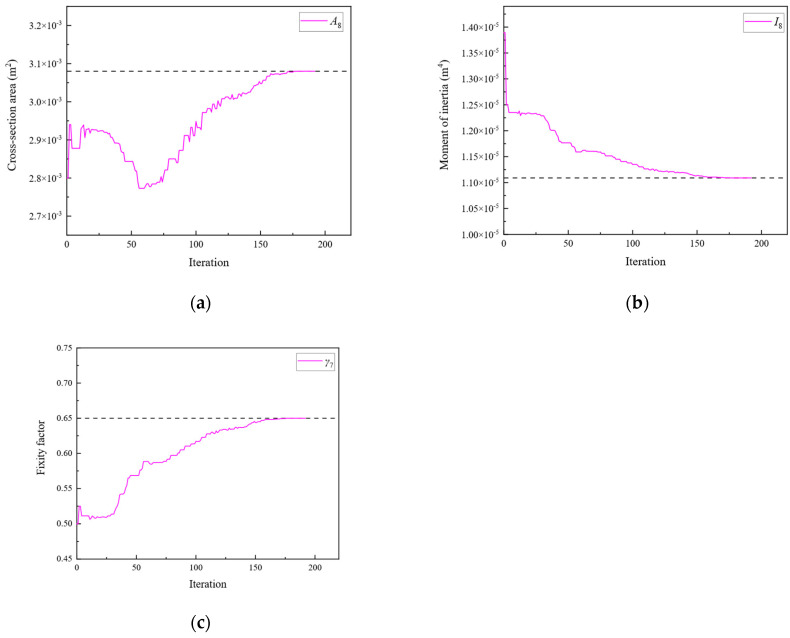
The variation of the parameters of Substructure 2 of the rigid frame with the number of iterations under Damage Scenario 3: (**a**) the cross-sectional area; (**b**) the moment of inertia; (**c**) the fixity factor.

**Figure 15:**
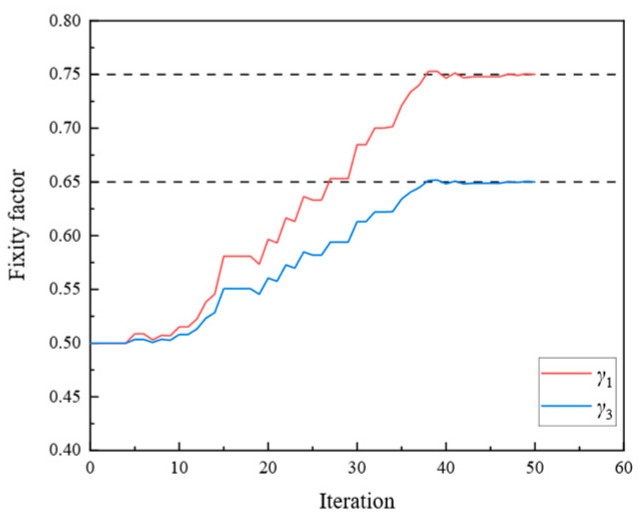
The variation of the parameters of Substructure 1 of the rigid frame with the number of iterations under Damage Scenario 4: the fixity factors.

**Figure 16:**
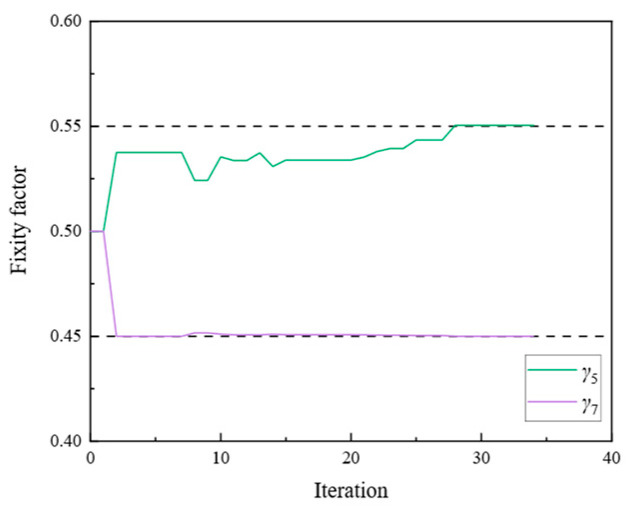
The variation of the parameters of Substructure 2 of the rigid frame with the number of iterations under Damage Scenario 4: the fixity factors.

Figure 9, Figure 10, Figure 11, Figure 12, Figure 13, Figure 14, Figure 15 and Figure 16 show the parameter iteration plots for the four damage scenarios listed in Table 1. Figure 9 and Figure 10 show the parameter iteration plots of Substructures 1 and 2, respectively, under Damage Scenario 1. Figure 11 and Figure 12 show the parameter iteration plots of Substructures 1 and 2, respectively, under Damage Scenario 2. Similarly, Figure 13 and Figure 14 show the parameter iteration plots for Substructures 1 and 2 under Damage Scenario 3, respectively. Figure 15 and Figure 16 show the parameter iteration plots for Substructures 1 and 2, respectively, under Damage Scenario 4. As shown in Figure 9, Figure 10, Figure 11, Figure 12, Figure 13, Figure 14, Figure 15 and Figure 16, all the unknown parameters converged to the “as-is” condition accurately.

**Figure 17:**
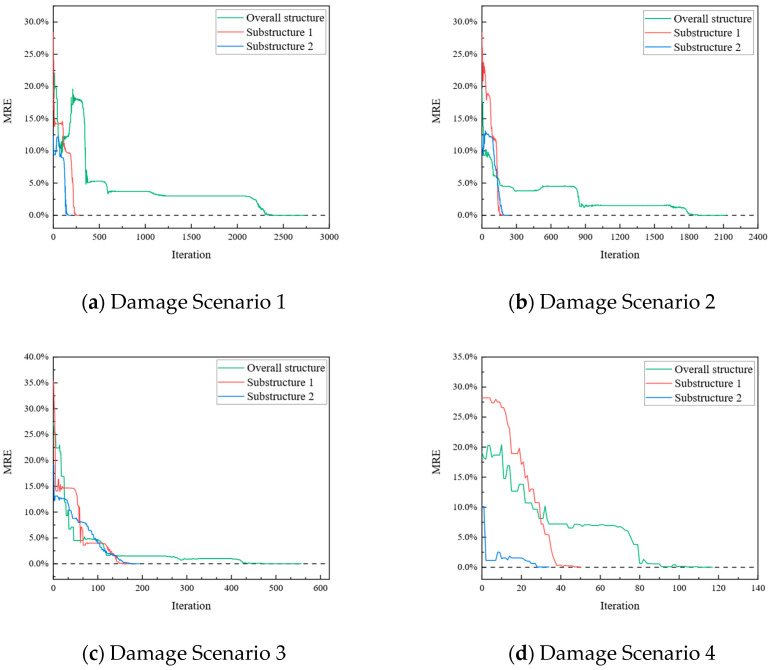
MRE results.

To compare the parameter identification of the overall structure with that of its substructures more effectively, their MRE values were calculated separately using Equation (6). Figure 17 illustrates the MRE values of all parameters for both overall structure identification and substructure identification under the four different damage scenarios. Figure 17a–d show the MRE plots of the parameter iterations of the overall structure and its substructures under the four different damage scenarios. 

For both Substructures 1 and 2, under the same damage scenario, the parameter identification of the substructures required fewer iteration steps for convergence compared to the parameter identification of the overall structure. Because the substiffness matrix had lower-order dimensions and fewer unknown parameters, the computational cost was reduced. The iterative values closely matched the actual values, with negligible errors. This indicates the effectiveness and accuracy of the parameter identification method proposed in Section 4.

## 5. Example of a Large and Complex Rigid Frame

**Figure 18:**
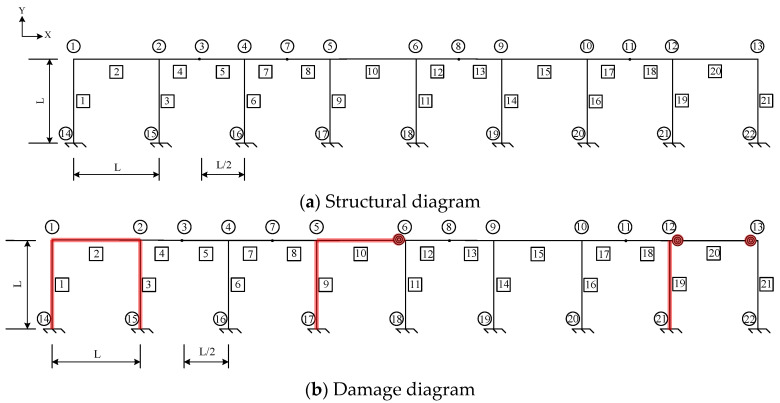
Multispan rigid frame.

**Figure 19:**
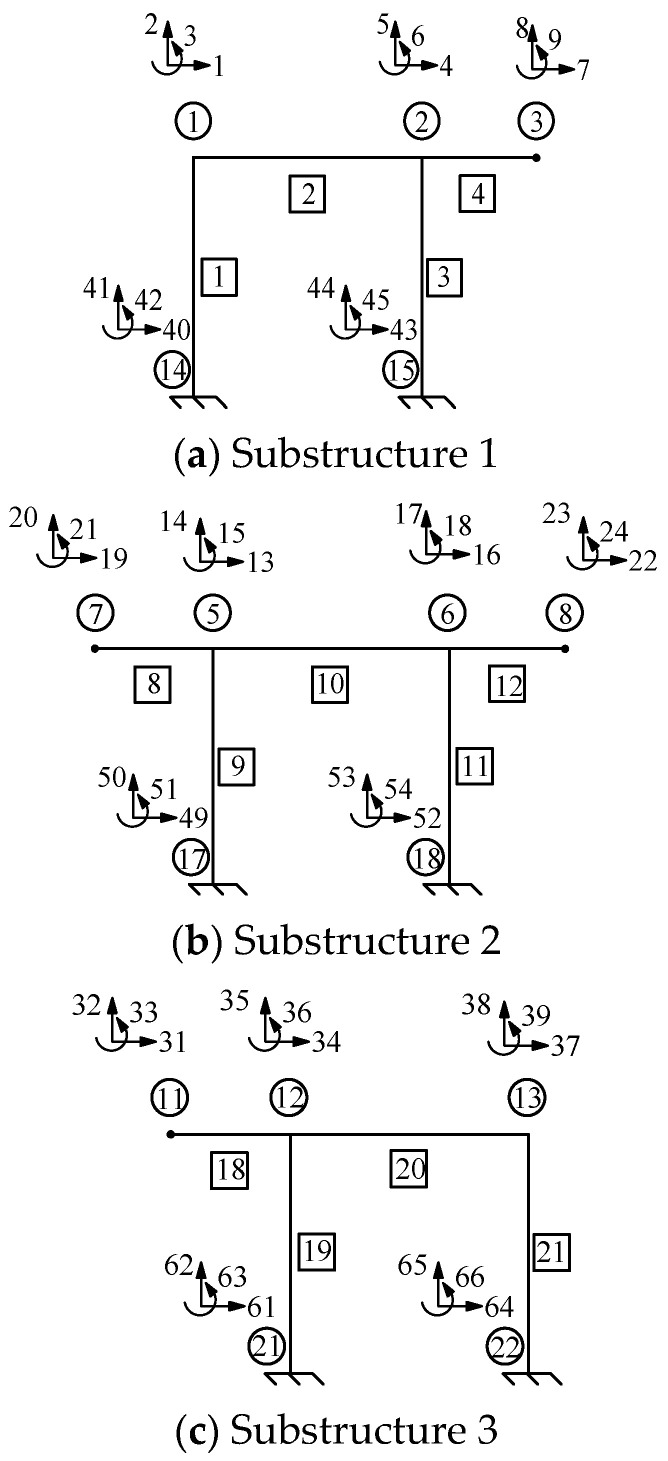
Schematic diagram of the rigid frame’s substructure.

The proposed stiffness separation method was evaluated by applying it to a multispan rigid frame (Figure 18). The frame’s modulus of elasticity was 206 GPa. For the “as-built” conditions, all the members featured the same cross-sectional area *A* = 5.6 × 10^−3^ m^2^, *I* = 2.779 × 10^−5^ m^4^, and *L* = 8 m, and a fixity factor of 1. The depth-to-span ratio (*h*/*L*) of the structure was 0.025. The “as-is” conditions are unknown and need to be determined when the rigid frame is damaged. Figure 18b shows the locations of the damaged members and joints, and the structural damage is indicated by the elements or joints highlighted in red. Table 3 lists the parameters that need to be identified and their corresponding values. The units of *A* and *I* are m^2^ and m^4^, respectively. Based on the locations of the structural damage, the multispan rigid frame structure was segmented into three substructures, as shown in Figure 19. Substructures 1, 2, and 3 are illustrated in Figure 19a, Figure 19b, and Figure 19c, respectively.

In this case, for each separated frame structure, independent forces were applied to ensure the load’s static responses. In reference to Figure 19a, when identifying Substructure 1, Node 3 is used to separate Substructure 1 from the overall structure, and its measured displacements under 7, 8, and 9 DOFs were taken as the separation boundary conditions. To obtain the responses of the structure, a horizontal external load of 20 kN was applied to Node 1, toward the right. Its measured displacements under 1, 2, 3, 4, 5, and 6 DOFs were used to establish an objective function.

In reference to Figure 19b, when identifying Substructure 2, Nodes 7 and 8 are used to separate Substructure 2 from the overall structure, and its measured displacements under 19, 20, 21, 22, 23, and 24 DOFs were considered the separation boundary conditions. To obtain the responses of the structure, a horizontal external load of 20 kN toward the right and a vertical external load of 20 kN downward were applied at Node 7. Additionally, the measured displacements under 13, 14, 15, 16, 17, and 18 DOFs were used to establish the objective function. 

In reference to Figure 19c, when identifying Substructure 3, Node 11 is used to separate Substructure 3 from the overall structure, and its measured displacements under 31, 32, and 33 DOFs were set as the separation boundary conditions. A horizontal external load of 20 kN toward the right was applied at Node 13. Subsequently, the objective function was established using the six measured displacements under 34, 35, 36, 37, 38, and 39 DOFs.

**Figure 20:**
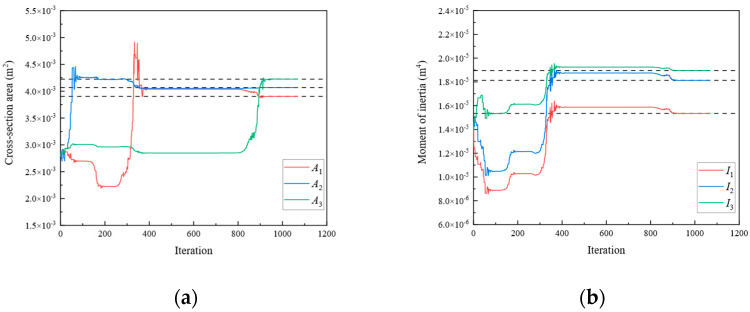
The variation of the parameters of Substructure 1 with the number of iterations: (**a**) the cross-sectional area; (**b**) the moment of inertia.

**Figure 21:**
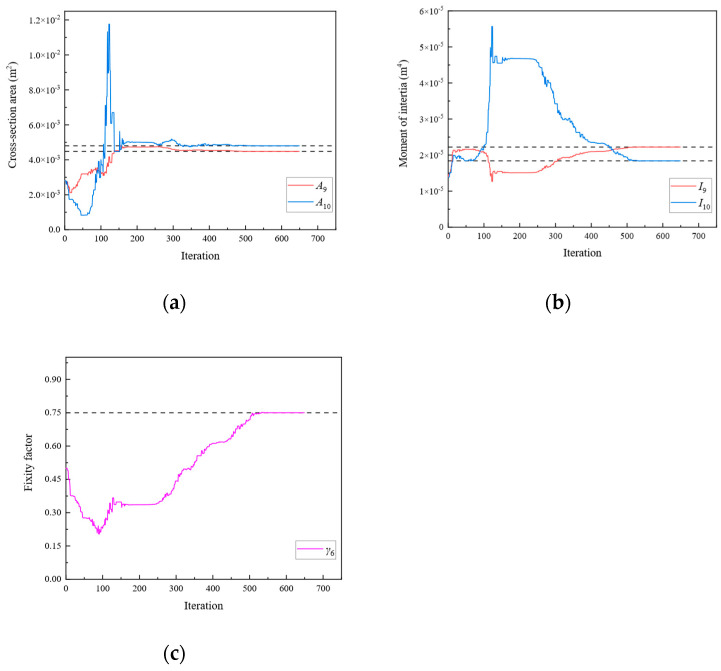
The variation of the parameters of Substructure 2 with the number of iterations: (**a**) the cross-sectional area; (**b**) the moment of inertia; (**c**) the fixity factor.

**Figure 22:**
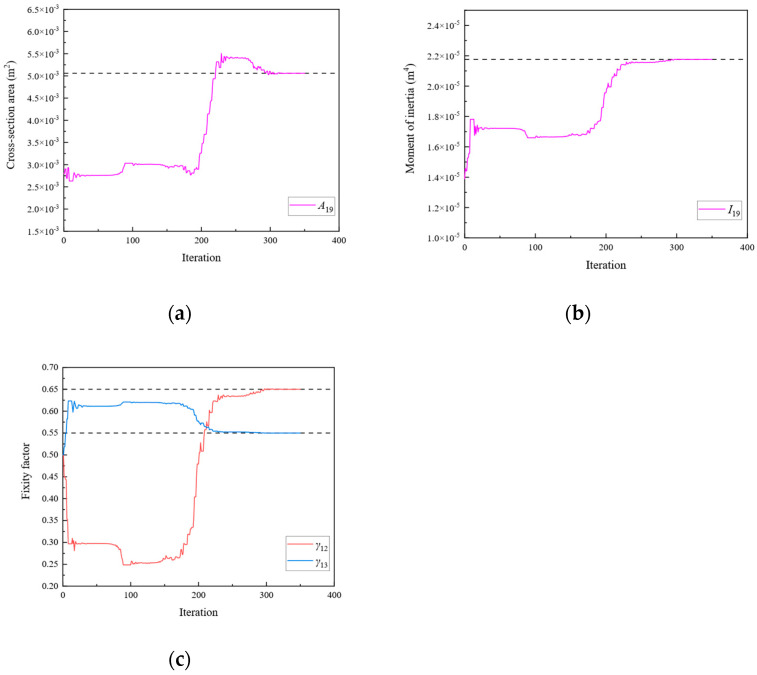
The variation of the parameters of Substructure 3 with the number of iterations: (**a**) the cross-sectional area; (**b**) the moment of inertia; (**c**) the fixity factor.

**Figure 23:**
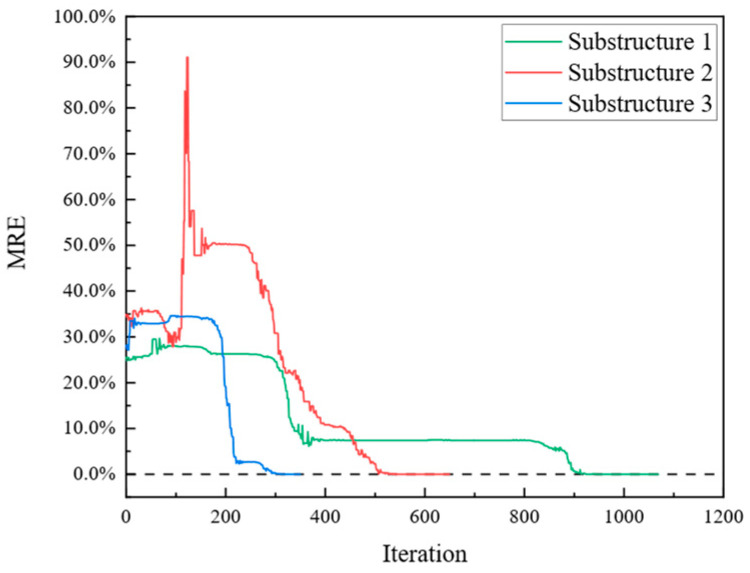
Trends of MRE.

Subsequently, optimization was performed for each of the three objective functions. The parameter iteration results for Substructures 1, 2, and 3 are shown in Figure 20, Figure 21 and Figure 22, respectively. The MRE values of parameter identification for each of the three substructures are shown in Figure 23. 

As shown in Figure 20, Figure 21, Figure 22 and Figure 23, Substructures 1, 2, and 3 were subjected to 1069, 649, and 350 iterations, respectively. Their objective functions converged, and their parameter identification errors were almost negligible. Table 4 presents a comparison of the iteration steps for Substructures 1, 2, and 3.

According to Table 4, each substructure has the same number of damaged elements, but their iterations for parameter convergence showed substantial discrepancies. The reason for this is that there exists a difference in the total number of unknown parameters for those three substructures. Consequently, the convergence of the objective function resulted in a different number of optimization iterations. However, successful identification was achieved, and the errors were negligible. This indicates the effectiveness and accuracy of the parameter identification method proposed in Section 4.

## 6. Conclusions

A stiffness separation method for the nondestructive static parameter identification of multispan rigid frame structures was introduced herein. The stiffness separation method utilizes the displacements at separation points as boundary conditions and transforms the complex problem of damage identification in structures into a simple calculation problem involving low-dimensional matrices. The feasibility, effectiveness, and accuracy of the proposed method were validated based on two examples. By segmenting a multispan rigid frame into multiple substructures and identifying each substructure separately, this method reduces the number of unknown parameters that need to be identified for each case. Additionally, for the large-scale structure, the forces are applied to each separated substructure to ensure obvious local static responses. Hence, this method reduces the stiffness matrix of the structure, enhances its operational efficiency, and facilitates its implementation. This method provides a new reference for the parameter identification of large-scale engineering structures. 

## Figures and Tables

**Table 1:** Damage scenarios of the three-span single-layer rigid frame.

Damage Scenario	Damage Parameter	Value	Damage Parameter	Value
1	*A*_1_	2.792 × 10^−3^	*I*_1_	8.053 × 10^−6^
	*A*_4_	3.080 × 10^−3^	*I*_4_	1.109 × 10^−5^
	*A*_7_	3.416 × 10^−3^	*I*_7_	1.344 × 10^−5^
	*A*_10_	3.584 × 10^−3^	*I*_10_	1.477 × 10^−5^
2	*A*_2_	2.792 × 10^−3^	*I*_2_	8.053 × 10^−6^
	*A*_3_	3.080 × 10^−3^	*I*_3_	1.109 × 10^−5^
	*A*_8_	3.416 × 10^−3^	*I*_8_	1.344 × 10^−5^
	*A*_9_	3.584 × 10^−3^	*I*_9_	1.477 × 10^−5^
3	*A*_3_	2.792 × 10^−3^	*I*_3_	8.053 × 10^−6^
	*A*_8_	3.080 × 10^−3^	*I*_8_	1.109 × 10^−5^
	*γ*_1_	0.75	*γ*_7_	0.65
4	*γ*_1_	0.75	*γ*_5_	0.55
	*γ*_3_	0.65	*γ*_7_	0.45

**Table 2:** Comparison of the iteration steps of the three-span single-layer rigid frame.

Damage Scenario	Damage Elements	Member Damage	Joint Damage	Unknown Parameters	Iterations
Scenario 1	4	4	0	8	2738
Scenario 2	4	4	0	8	2118
Scenario 3	4	2	2	6	557
Scenario 4	4	0	4	4	117

**Table 3:** Damage scenarios of the multispan rigid frame.

Substructure	Damage Parameter	Value	Damage Parameter	Value
1	*A*_1_	3.904 × 10^−3^	*I*_1_	1.535 × 10^−5^
	*A*_2_	4.068 × 10^−3^	*I*_2_	1.813 × 10^−5^
	*A*_3_	4.224 × 10^−3^	*I*_3_	1.896 × 10^−5^
2	*A*_9_	4.480 × 10^−3^	*I*_9_	2.223 × 10^−5^
	*A*_10_	4.800 × 10^−3^	*I*_10_	1.840 × 10^−5^
	*γ*_6_	0.75		
3	*A*_19_	5.060 × 10^−3^	*I*_19_	2.176 × 10^−5^
	*γ*_12_	0.65	*γ*_13_	0.55

**Table 4:** Comparison of iteration steps of Substructures 1–3.

Damage Scenario	Damage Elements	Member Damage	Joint Damage	Unknown Parameters	Iterations
Substructure 1	3	3	0	6	1069
Substructure 2	3	2	1	5	649
Substructure 3	3	1	2	4	350

## Data Availability

Data is contained within the article.
